# Supplementary material for: A rapid simple point-of-care assay for the detection of SARS-CoV-2 neutralizing antibodies
Source: Commun Med (Lond). 2021 Nov 11;1:46. doi: 10.1038/s43856-021-00045-9 (PMC9053278; doi:10.1038/s43856-021-00045-9)
Supplement: Supplementary file 1 — Supplemental Materials [file 43856_2021_45_MOESM1_ESM.docx]

**Rapid detection of SARS-CoV-2 neutralizing antibodies at the point-of-care by cellulose-based assay**

Patthara Kongsuphol^1,*^, Huan Jia^1,*^, Hoi Lok Cheng^1,*^, Yue Gu^2,*^,  Bhuvaneshwari D/O Shunmuganathan^2,*^, Ming Wei Chen^3^, Sing Mei Lim^1^, Say Yong Ng^1^, Paul Ananth Tambyah^4^, Haziq Nasir^4^, Xiaohong Gao^3^, Dousabel Tay^5^, Seunghyeon Kim^5^, Rashi Gupta^2^, Xinlei Qian^6^, Mary M Kozma^6^, Kiren Purushotorman^2^, Megan E McBee^1^, Paul A MacAry^2,6,#^, Hadley D Sikes^1,5,#^, Peter R Preiser^1,3,#^

^1^Antimicrobial Resistance Interdisciplinary Research Group (AMR-IRG), Singapore-MIT Alliance in Research and Technology (SMART), #03-10/11 Innovation Wing, 1 CREATE way, Singapore 138602

^2^Department of Microbiology and Immunology, Yong Loo Lin School of Medicine, National University of Singapore (NUS), 5 Science Drive 2, Blk MD4, Level 3, Singapore 117545.

^3^School of Biological Science (SBS), Nanyang Technological University (NTU), 60 Nanyang Dr, Singapore 637551

^4^Department of Medicine, National University Hospital (NUH), 5 Lower Kent Ridge Rd, Singapore 119074.

^5^Department of Chemical Engineering, Massachusetts Institute of Technology (MIT), 25 Ames Street, Building 66 Cambridge, MA 02139, USA

^6^Life Sciences Institute (LSI), National University of Singapore (NUS), Center for Life Sciences, #05-02, 28 Medical Drive, Singapore 117456

*PK, HJ, HC, YG and BD/OS contribute equally as co-first authors.

^#^PAM, HDS and PRP are co-corresponding authors. Emails: [micpam@nus.edu.sg](mailto:micpam@nus.edu.sg); [sikes@mit.edu](mailto:sikes@mit.edu); [prpreiser@ntu.edu.sg](mailto:prpreiser@ntu.edu.sg).

**Supplementary Table 1. Patient Demographic information**

| Sample | Severity | No. days post admission | | | No. of days of symptoms before admission | Remark |
| --- | --- | --- | --- | --- | --- | --- |
|  |  | **FV1 (Follow up visit)** | **FV2** | **FV3** |  |  |
| P01 | Mild | 38 |  |  | 7 |  |
| P02 | Moderate | 50 |  |  | 6 |  |
| P04 | Mild | 29 | 114 | 196 | 14 |  |
| P06 | Mild | 30 | 99 | 183 | 8 |  |
| P07 | Severe | 36 |  |  | 5 |  |
| P08 | Severe | 36 |  |  | 7 |  |
| P09 | Mild | 36 |  |  | 3 |  |
| P10 | Mild | 35 |  |  | 4 |  |
| P11 | Mild | 33 |  |  | NA | Asymptomatic |
| P13 | Mild | 51 | 82 | 194 | -9 | Symptom started on Day 9 post admission |
| P14 | Mild | 48 |  |  | NA | Asymptomatic |
| P15 | Mild | 38 | 129 | 192 | 12 |  |
| P19 | Mild | 53 | 92 | 192 | -3 | Symptom started on Day 3 post admission |
| P20 | Mild | 44 | 93 | 191 | 0 |  |
| P21 | Severe | 59 | 121 | 213 | 0 |  |
| P22 | Mild | 54 | 100 | 199 | 0 |  |
| P23 | Mild | 67 | 107 | 190 | -7 | Symptom started on Day 7 post admission |
| P24 | Mild | 73 |  |  | 0 |  |
| P25 | Severe | 44 |  |  | 0 |  |
| P30 | Mild | 51 | 98 |  | 30 |  |
| P37 | Mild | 60 | 101 |  | 0 |  |
| P42 | Mild | 64 | 103 | 196 | NA | Asymptomatic |
| P45 | Mild | 50 |  |  | 7 |  |
| P48 | Mild | 55 | 108 |  | -8 | Symptom started on Day 8 post admission |

**Supplementary Table 2a. Accuracy of surrogate virus neutralization test (sVNT) as compared to pseudovirus neutralization test (pVNT)**

| Statistic | Value | 95% CI |
| --- | --- | --- |
| Sensitivity | 90.0% | 55.5% to 99.8% |
| Specificity | 86.5% | 71.2% to 95.5% |
| Positive Predictive Value* | 64.3% | 43.7% to 80.7% |
| Negative Predictive Value* | 97.0% | 83.2% to 99.5% |
| **Accuracy*** | **87.2%** | **74.3% to 95.2%** |

**Supplementary Table 2b. Accuracy of cellulose pull down (cpVNT) as compared to pseudovirus neutralization test (pVNT)**

| Statistic | Value | 95% CI |
| --- | --- | --- |
| Sensitivity | 80.0% | 44.4% to 97.5% |
| Specificity | 84.4% | 67.2% to 94.7% |
| Positive Predictive Value* | 61.5% | 40.31% to 79.1% |
| Negative Predictive Value* | 93.1% | 79.5% to 97.9% |
| **Accuracy*** | **83.3%** | **68.6% to 93.0%** |

**Supplementary Table 2c. Accuracy of cellulose pull down virus neutralization test (cpVNT) as compared to surrogate virus neutralization test (sVNT)**

| Statistic | Value | 95% CI |
| --- | --- | --- |
| Sensitivity | 85.7% | 57.2% to 98.2% |
| Specificity | 96.6% | 82.2% to 99.9% |
| Positive Predictive Value* | 92.3% | 63.4% to 98.8% |
| Negative Predictive Value* | 93.3% | 79.5% to 98.1% |
| **Accuracy*** | **93.0%** | **80.9% to 98.5%** |

* These values depend on the prevalence of the disease. The prevalence is calculated from the sample size. It may not reflect the real disease prevalence.

**Supplementary Table 3. Comparison chart between different VNTs**

**Supp. Table 1. Patient Demographic information (cont.)**

| **Criteria** | **cVNT** | **pVNT** | **sVNT** | **cpVNT** |
| --- | --- | --- | --- | --- |
| **Assay time** | ✓ 4 days | ✓ 1 day | ✓✓ 2 hours | ✓✓✓ 10 min |
| **Sample processing** | ✓✓ Yes | ✓✓ Yes | ✓✓ Yes | ✓✓ Yes |
| **Requirement of skillful personnel** | ✓✓ Yes | ✓✓ Yes | ✓✓✓ No | ✓✓✓ No |
| **Lab equipment** | ✓✓ Yes | ✓✓ Yes | ✓✓ Yes | ✓✓✓ No |
| **Biosafety requirement** | ✓ BSL-3 | ✓✓ BSL-2 | ✓✓ BSL-2 | ✓✓✓ No |
| **Ease of mass manufacture** | ✓ No | ✓ No | ✓✓✓ Yes | ✓✓✓ Yes |
| **Production cost** | ✓✓ Medium | ✓✓ Medium | ✓✓✓ Low | ✓✓✓Low |

✓ Less preferred criteria; ✓✓ medium preferred criteria; ✓✓✓ most preferred criteria

More preferred

Less preferred


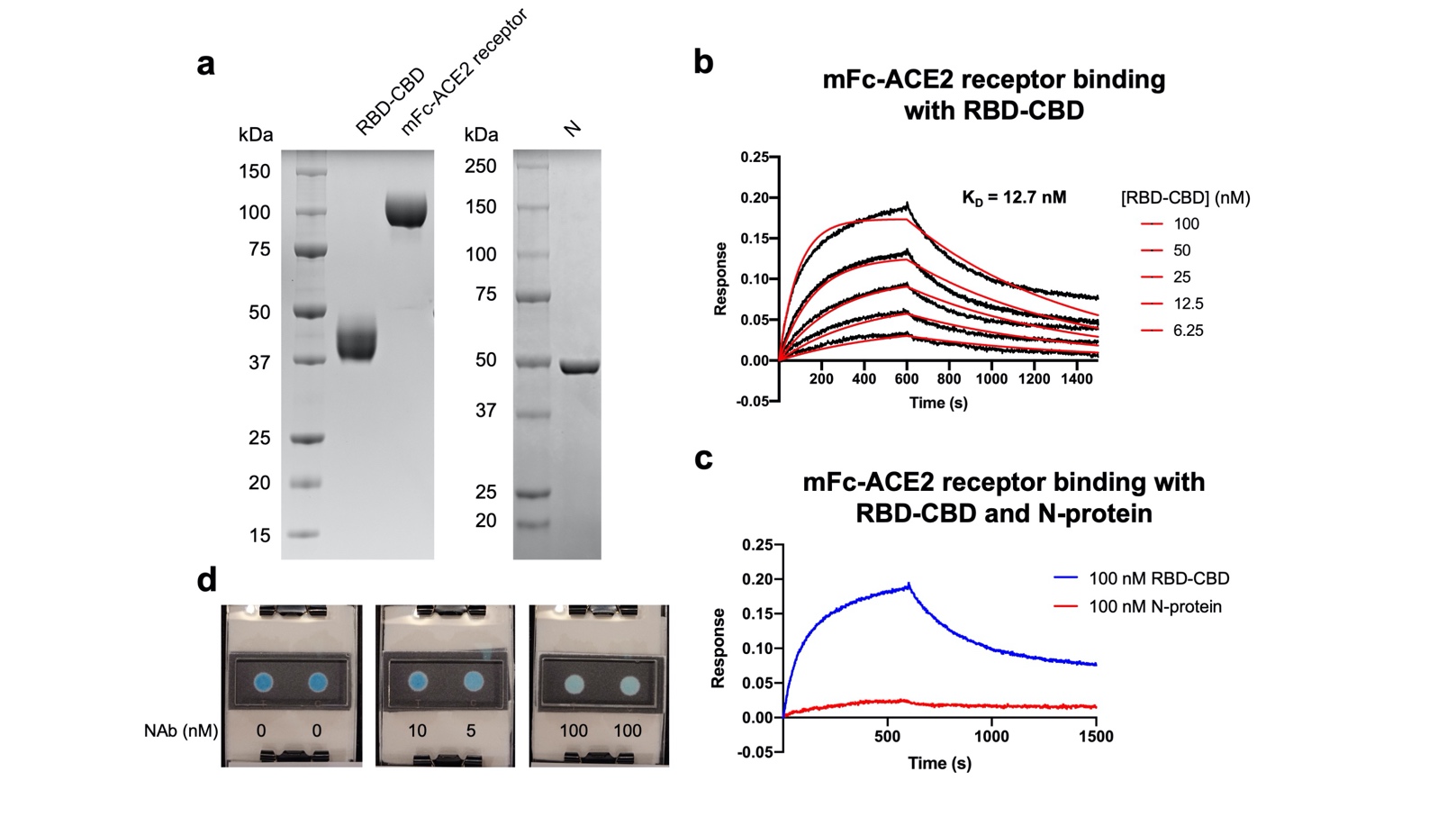


**Supplementary Fig. 1. Receptor binding domain (RBD) fused cellulose binding domain (RBD-CBD) and monoFc (mFc) fused angiotensin converting enzyme 2 receptor (mFc-ACE2) purifications and kinetic study and example of images obtained from light box and phone camera. (a)** Sodium dodecyl sulphate–polyacrylamide gel electrophoresis (SDS-PAGE) images of recombinant RBD-CBD, mFc-ACE2 receptor and nucleocapsid (N) proteins expressed and purified for this study. **(b)** Bio-layer Interferometry (BLI) of biotinylated mFc-Ace2 receptor on streptavidin probes and different concentrations of RBD-CBD ranging from 6.25 nM – 100 nM. K_D_ value was observed at 12.7 nM. **(c)** BLI of biotinylated mFc-Ace2 receptor on streptavidin probes with 100 nM RBD-CBD and N protein from SARS-CoV-2. **(d)** Examples of images obtained from the phone camera and the light box. Each cellulose testing unit was tested using different neutralizing antibody (NAb) concentrations spiked in healthy control plasma, including 0 nM in the left unit, 10 and 5 nM in the middle unit, and 100 nM in the right unit. Optimized cellulose pull down virus neutralization test (cpVNT) testing condition was used to perform these tests.


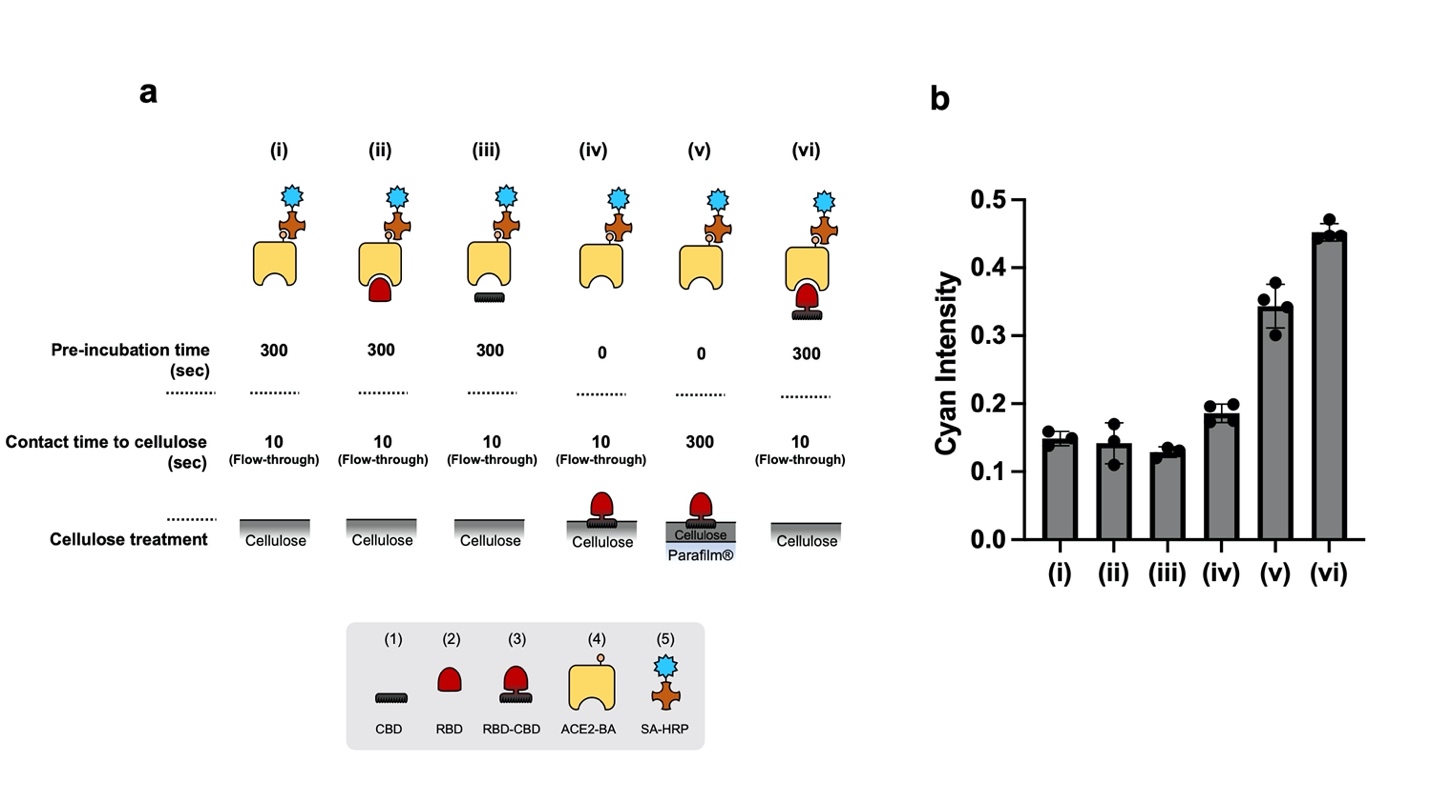


**Supplementary Fig. 2. Comparison of signals on cellulose-based vertical flow device using different test configurations.** Inset depicts drawings of (1) cellulose binding domain (CBD), (2) receptor binding domain (RBD), (3) RBD tagged CBD (RBD-CBD), (4) angiotensin converting enzyme 2 receptor (ACE2) conjugated biotin (ACE2-BA) and (5) horse radish peroxidase (HRP) conjugated streptavidin (SA-HRP). **(a)** Different test configurations were tested to ensure that signals detected from RBD-CBD/ACE2-BA-SA-HRP complex were specific, and integration of RBD-CBD promotes rapid signal detection. **(b)** Cyan intensities measured from each test configuration are in presented in a bar chart format. Based on the Bio-layer Interferometry (BLI) data (**Supplementary Fig. 5B**), >80% of RBD-CBD/ACE2 complex was formed within 300 sec (5 min). Therefore 300 sec incubation time were used to allow RBD-CBD/ACE2-BA-SA-HRP to form for the cellulose-based vertical flow device. **(i)** ACE2-BA-SA-HRP alone or with **(ii)** bare RBD or **(iii)** bare CBD produced equally low signals of cyan intensities. These results indicated that bare RBD alone could not be immobilized on the cellulose matrix within the 10 second flow-through time. In addition, CBD does not produce non-specific signal between ACE2-SA-HRP and CBD. **(iv)** Equivalent amount of RBD-CBD to the liquid phase premix conditions was immobilized on cellulose matrix. Data demonstrated that only slight increase in cyan intensity was observed as compared to the baseline value (shown in A). This data indicated that the 10 second flowthrough time was not sufficient to allow the complex formation. **(v)** In a similar configuration to (iv), longer incubation time between RBD-CBD and ACE2-BA-SA-HRP allowed significant increase in cyan intensity, indicating that the complex formed much more efficient with longer incubation time. However, pre-immobilizing of RBD-CBD on cellulose paper introduced complicated workflow to the assay in which solution must be maintained on the cellulose surface for 5 min before it is allowed to flow pass the test spot. **(vi)** Due to the known property of CBD which can interact rapidly to cellulose matrix^1^, the pre-mix condition was introduced to allow RBD-CBD/ACE2-BA-SA-HRP complex formation before the whole complex can be captured onto the cellulose paper. Cyan intensity from this configuration showed the highest value as compared to others, confirming that CBD can be captured rapidly onto the cellulose matrix. In addition, cyan intensity from condition (vi) has shown to be significantly higher than condition (v) (Student *t*-test, *p*-value of 0.00076), suggesting that the liquid phase incubation promoted more efficient complex formation as compared to the pre-immobilized capture reagent at equimolar concentration. Each data point were represented as mean ± standard deviation (SD) and was performed at least in triplicates.


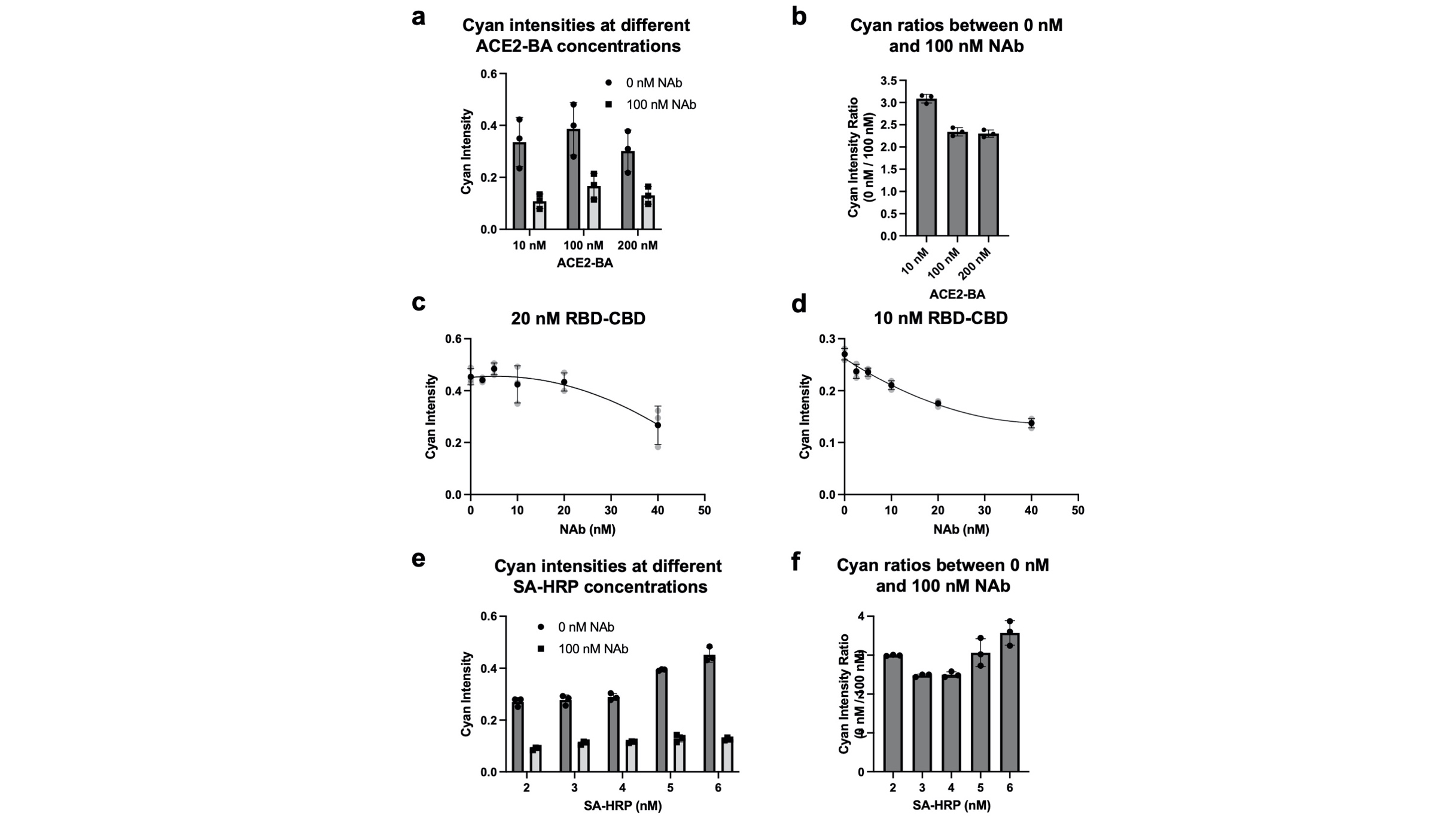


**Supplementary Fig. 3. Optimization of receptor binding domain (RBD) fused cellulose binding domain (RBD-CBD), biotin (BA) conjugated angiotensin converting enzyme 2 receptor (ACE2-BA) and streptavidin (SA) conjugated horse radish peroxidase (SA-HRP) concentrations for cellulose pull down virus neutralization test (cpVNT).**  To optimize for reagent concentrations, plasma samples containing 0 or 100 nM neutralizing antibodies (NAb) were used to determine the maximal (max) and minimal (min) cyan intensity signals. Concentrations of reagents that provide the highest ratio of max/min (0 nM:100 nM) signals were selected for the cpVNT. **(a)** Cyan intensity obtained from different RBD-CBD concentrations when ACE2-BA and SA-HRP concentrations were fixed at 20 nM and 2 nM, respectively. **(b)** Ratio of cyan intensity obtained from 0 nM:100 nM NAb. Highest ratio was observed from 10 nM of ACE2-BA, therefore this concentration was selected. Two different concentrations of RBD-CBD were tested including 10 and 20 nM. Concentrations of ACE2-BA and SA-HRP were fixed at 10 nM and 2 nM, respectively. The ratio between 0 nM:100 nM NAb of both RBD-CBD concentrations show similar value at ~2.1, therefore different concentrations of NAb were tested to further inspect changes in cyan intensities. Cyan signals obtained from **(c)** 20 nM RBD-CBD showed minimal changes in the color intensities at low NAb concentrations whereas the signals obtained from **(d)** 10 nM RBD-CB showed distinguishable signals at low NAb concentrations, therefore 10 nM RBD-CBD was selected for cpVNT. To optimize for SA-HPR concentration, RBD-CBD and ACE2-BA concentrations were fixed at 10 nM. **(e)** Cyan intensities obtained different concentrations of SA-HRP showed minimal changes at 100 nM NAb. More changes were observed from 0 nM NAb where 6 nM SA-HRP show the highest signal. **(f)** Ratios between 0 nM: 100 nM NAb obtained from different concentrations of SA-HRP. Highest ratio was observed at 6 nM SA-HRP, therefore this concentration was selected for cpVNT. All data were performed in triplicates and represented as mean ± standard deviation (SD).

**
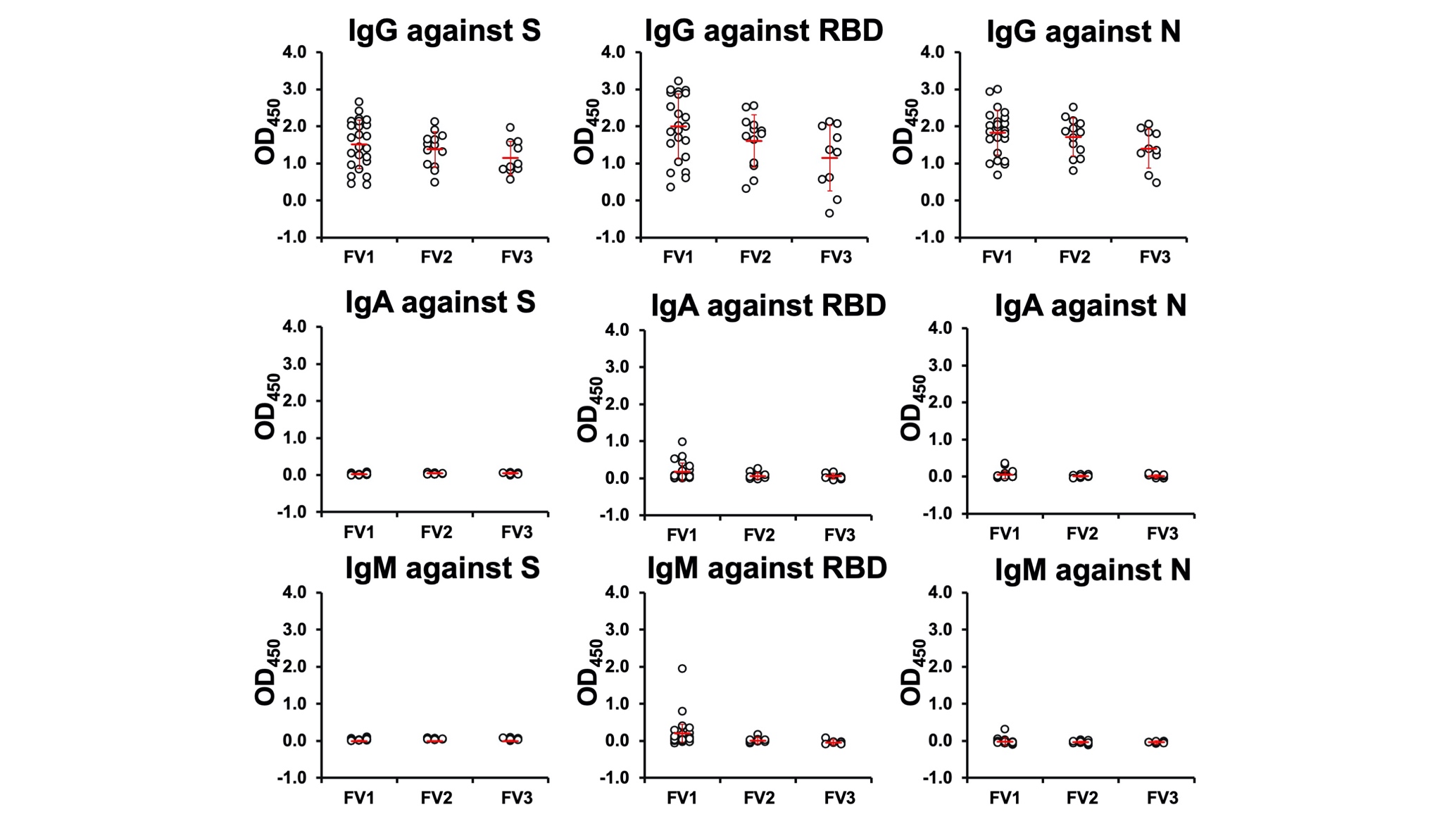
**

**Supplementary Fig. 4.** **Detection of immunoglobulin (Ig) isotype G (IgG), A (IgA) and and M (IgM) against SARS-CoV-2 Spike (S), receptor binding domain (RBD) and nucleocapsid (N) proteins using enzyme-link immunosorbent assay (ELISA).** Plasma samples were obtained from COVID-19 convalescent patients at different follow up visits (FV) post infections. FV1, 2, and 3 were ranged from 29-73 days, 82-129 days and 183-213 days, respectively. Each data point represented mean values of optical signal read at 450 nm. Each point was performed in triplicates. Overall mean ± standard deviation (SD) values from different samples from each visits were shown in red lines


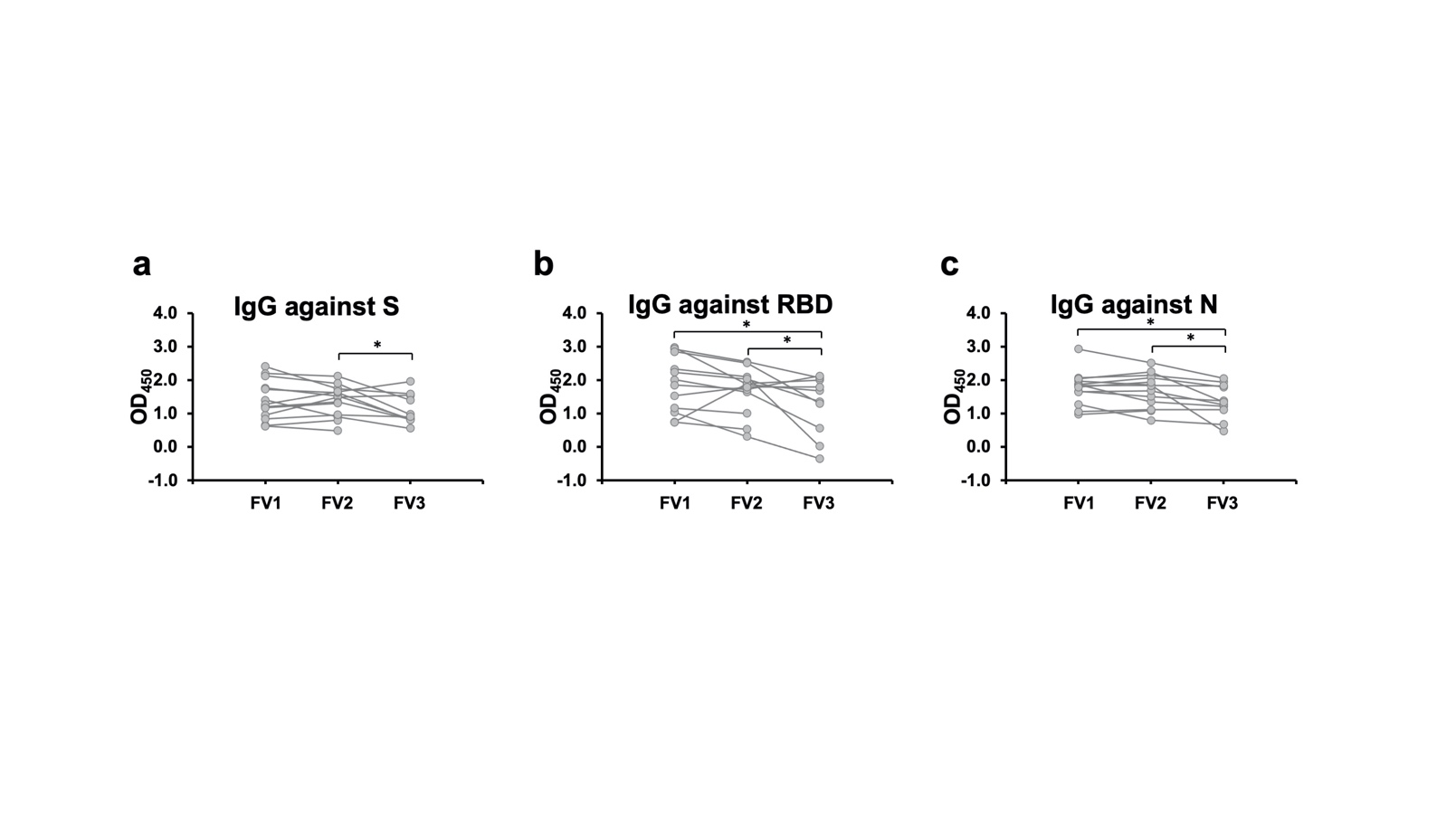


**Supplementary Fig. 5.** **Assessment of immunoglobulin (Ig) isotype G (IgG) against different SARS-CoV-2 antigens.** Assessment of IgG against **(a)** spike (S), **(b)** receptor binding domain (RBD) and **(c)** nucleocapsid (N) proteins from plasma samples obtained from confirmed COVID-19 patients at different visits. Only samples from patients who came for the follow up visits were included in this analysis. Sample sizes for follow up visit (FV) 1 (FV1), FV2 and FV3 were 13, 13 and 10, respectively. Statistical analysis was done using student pair *T*-test. * indicates statistical difference at *p* value of 0.002. Each data point represented mean values of optical signal read at 450 nm. Each point was performed in triplicates. Lines connecting between different data point indicated that signals were obtained from the same sample from different visits.


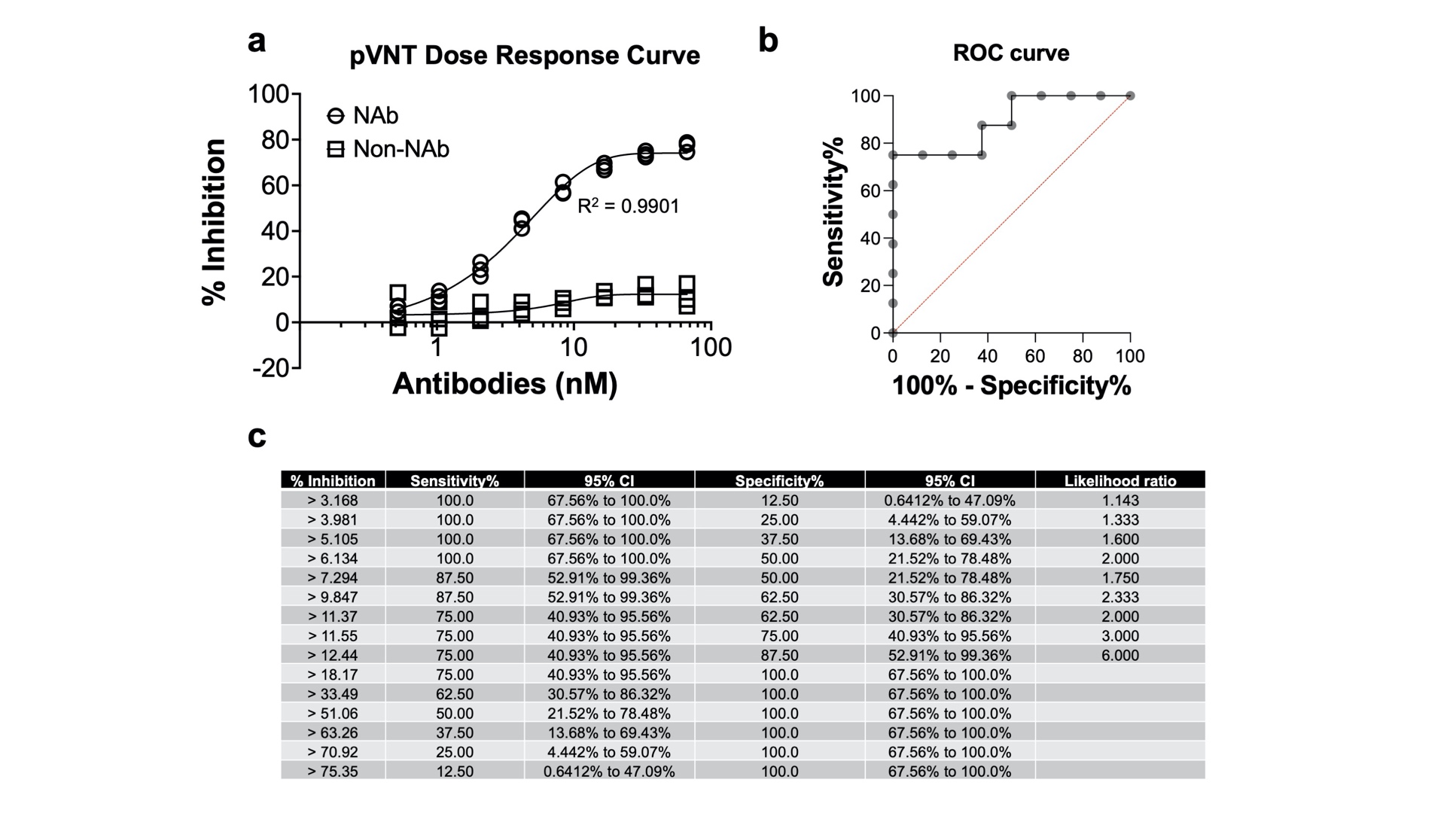


**Supplementary Fig. 6.** **Analysis of surrogate virus neutralization test (sVNT) performance using known concentrations of antibodies to define a cut-off inhibitory percentage**. **(a)** Dose response curves of SARS-CoV-2 nutralizing antibodies (Nabs) and hAnti-receptor binding domain (RBD) non-neutralizing antibodies spiked in human plasma. Each data point represented mean ± standard deviation (SD). Each point was performed at least in triplicates. 4 Parameters logistic model was used to draw the fitted curve with R^2^ value of 0.9901. (**b)** Receive operating characteristic (ROC) curve and **(c**) sensitivity and specific of sVNT at different signal inhibitory percentages. At ~20% inhibitory percentage, sVNT sensitivity maintains high at 75% and achieve specificity of 100%. As such, 20% cut-off is employed for sVNT.

**
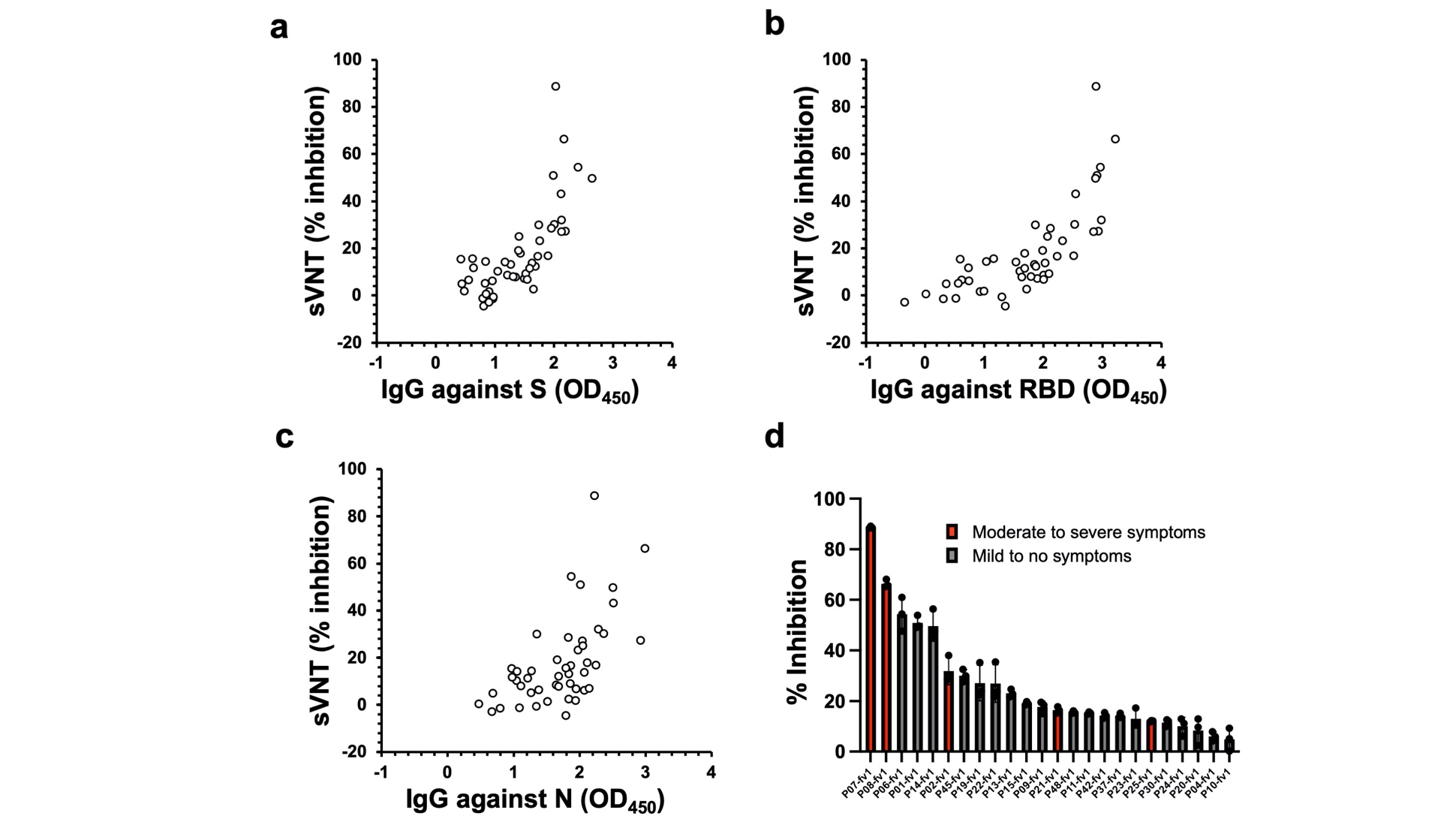
**

**Supplementary Fig. 7. Correlation between neutralizing antibodies (Nabs) measured using surrogate virus neutralizing test (sVNT) and immunoglobulin G (IgG) against different SARS-CoV2 proteins obtained from enzyme-link immunosorbent assay (ELISA).** Correlation of sVNT and IgG against **(a)** spike (S), **(b)** receptor binding domain (RBD) and **(c)** nucleocapsid (N) proteins. **(d)** NAbs status from follow up visit 1 (FV1) determined using sVNT. Data were arranged in the order of highest to lowest values. Red bars represent samples which exhibit moderate to severe symptoms whereas black bars represent samples which exhibit mild to no symptoms. Each data point in dot plots represented mean values of different samples. Each point was performed in triplicates. Data in bar chart were represented at mean ± standard deviation (SD). Each point was performed in triplicates.

**
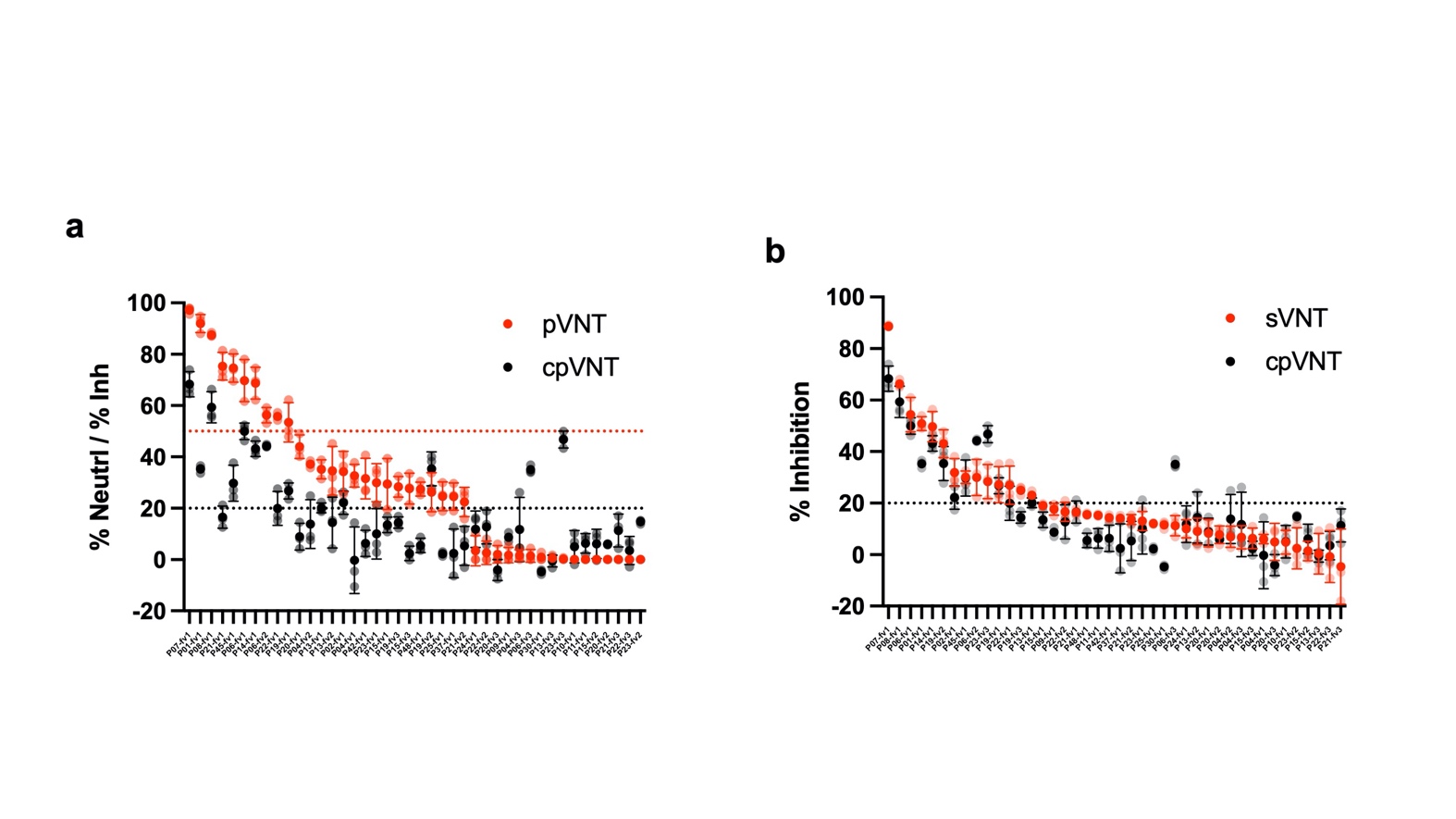
**

**Supplementary Fig. 8. Alternative correlation plots of cellulose pull down virus neutralization test (cpVNT) against pseudovirus neutralization test (pVNT) and surrogate virus neutralization test (sVNT).** **(a)** Correlation plot between pVNT and cpVNT with neutralizing antibody (NAb) status arranged from highest to lowest values. Red and black lines represent cut-off values which determine positive and negative NAb status of pVNT and cpVNT at 50% and 20%, respectively. **(b)** Alternative correlation plot of sVNT and cpVNT with NAb status arranged from highest to lowest values. Black line represents a cut-off value for sVNT and cpVNT at 20%. All data points were represented as mean ± standard deviation (SD). Each point was performed at least in triplicates.


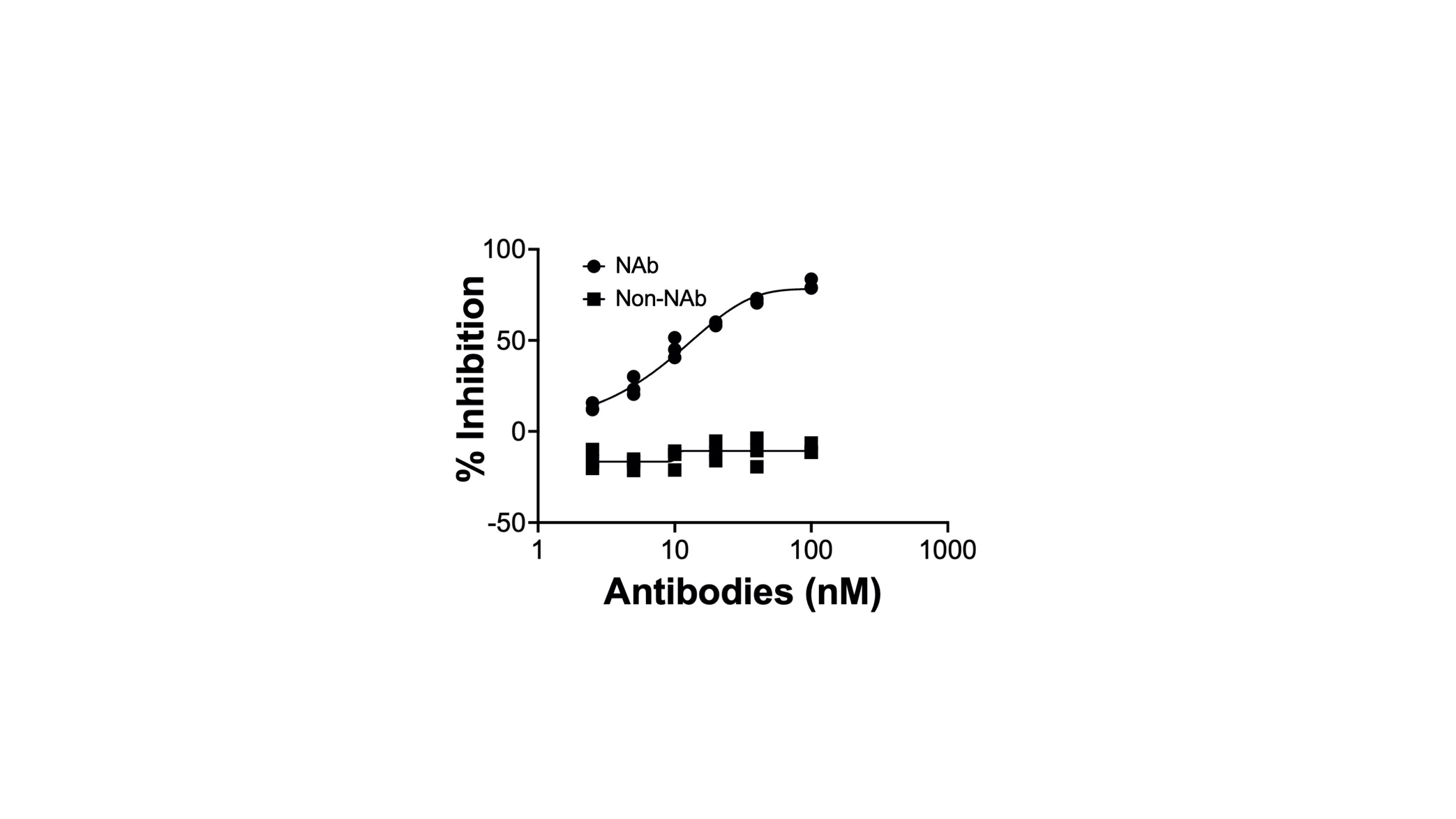


**Supplementary Fig. 9. Cellulose pull down virus neutralization test (cpVNT) results obtained from human serum samples.** Inhibitory percentages derived from cyan intensity signals obtained from different concentrations of mouse anti SARS-CoV-2 neutralizing antibodies and human anti spike subunit 1 (S1) antibodies that did not possess neutralizing property. Limit of detections (LOD) were determined using mean + 3-fold standard deviation (SD) formular and represented as red lines. All data points were represented as mean ± SD. Each point was performed at least in triplicates.
