# Supplementary material for: A rapid simple point-of-care assay for the detection of SARS-CoV-2 neutralizing antibodies
Source: Commun Med (Lond). 2021 Nov 11;1:46. doi: 10.1038/s43856-021-00045-9 (PMC9053278; doi:10.1038/s43856-021-00045-9)
Supplement: Supplementary file 3 — Description of Additional Supplementary Files [file 43856_2021_45_MOESM3_ESM.pdf]

Description of Additional Supplementary Files

File Name: Supplementary Data 1

Description: Source date for Fig 2C, Fig 3A-E and Fig 4A-F
