# Supplementary material for: A rapid simple point-of-care assay for the detection of SARS-CoV-2 neutralizing antibodies
Source: Commun Med (Lond). 2021 Nov 11;1:46. doi: 10.1038/s43856-021-00045-9 (PMC9053278; doi:10.1038/s43856-021-00045-9)
Supplement: Supplementary file 5 — Reporting Summary [file 43856_2021_45_MOESM5_ESM.pdf]

nature portfolio

Corresponding author(s): Paul McAry, Hadley Sikes, Peter Preiser

Last updated by author(s): 14 Oct 2021

Reporting Summary

Nature Portfolio wishes to improve the reproducibility of the work that we publish. This form provides structure for consistency and transparency in reporting. For further information on Nature Portfolio policies, see our Editorial Policies and the Editorial Policy Checklist.

Statistics

For all statistical analyses, confirm that the following items are present in the figure legend, table legend, main text, or Methods section.

n/a	Confirmed	
☐	☒	The exact sample size (n) for each experimental group/condition, given as a discrete number and unit of measurement
☐	☒	A statement on whether measurements were taken from distinct samples or whether the same sample was measured repeatedly
☐	☒	The statistical test(s) used AND whether they are one- or two-sided<br>*Only common tests should be described solely by name; describe more complex techniques in the Methods section.*
☐	☒	A description of all covariates tested
☐	☒	A description of any assumptions or corrections, such as tests of normality and adjustment for multiple comparisons
☐	☒	A full description of the statistical parameters including central tendency (e.g. means) or other basic estimates (e.g. regression coefficient) AND variation (e.g. standard deviation) or associated estimates of uncertainty (e.g. confidence intervals)
☒	☐	For null hypothesis testing, the test statistic (e.g. F, t, r) with confidence intervals, effect sizes, degrees of freedom and P value noted<br>*Give P values as exact values whenever suitable.*
☒	☐	For Bayesian analysis, information on the choice of priors and Markov chain Monte Carlo settings
☒	☐	For hierarchical and complex designs, identification of the appropriate level for tests and full reporting of outcomes
☐	☒	Estimates of effect sizes (e.g. Cohen's d, Pearson's r), indicating how they were calculated

Our web collection on statistics for biologists contains articles on many of the points above.

Software and code

Policy information about availability of computer code

Data collection	NA
Data analysis	NA

For manuscripts utilizing custom algorithms or software that are central to the research but not yet described in published literature, software must be made available to editors and reviewers. We strongly encourage code deposition in a community repository (e.g. GitHub). See the Nature Portfolio guidelines for submitting code & software for further information.

Data

Policy information about availability of data

All manuscripts must include a data availability statement. This statement should provide the following information, where applicable:

- Accession codes, unique identifiers, or web links for publicly available datasets
- A description of any restrictions on data availability
- For clinical datasets or third party data, please ensure that the statement adheres to our policy

Source data is available in Supplementary Data 1

March 2021

Field-specific reporting

Please select the one below that is the best fit for your research. If you are not sure, read the appropriate sections before making your selection.

☒ Life sciences ☐ Behavioural & social sciences ☐ Ecological, evolutionary & environmental sciences

For a reference copy of the document with all sections, see nature.com/documents/nr-reporting-summary-flat.pdf

Life sciences study design

All studies must disclose on these points even when the disclosure is negative.

Sample size	24 COVID-19 convalescent samples
Data exclusions	NA
Replication	Each data point obtained from each sample was performed in triplicates
Randomization	Randomization is not relevance to this study. Known COVID-19 positive subjects were recruited for this study to evaluate for their neutralizing antibodies using different virus neutralization tests.
Blinding	Status of neutralizing antibodies were unknown to researchers who ran surrogate virus neutralization test (sVNT) and cellulose pull down virus neutralization test (cpVNT) until all test results were obtained from all clinical samples.

Reporting for specific materials, systems and methods

We require information from authors about some types of materials, experimental systems and methods used in many studies. Here, indicate whether each material, system or method listed is relevant to your study. If you are not sure if a list item applies to your research, read the appropriate section before selecting a response.

Materials & experimental systems

n/a	Involved in the study	
☐	☒	Antibodies
☐	☒	Eukaryotic cell lines
☒	☐	Palaeontology and archaeology
☒	☐	Animals and other organisms
☐	☒	Human research participants
☒	☐	Clinical data
☒	☐	Dual use research of concern

Methods

n/a	Involved in the study	
☒	☐	ChIP-seq
☒	☐	Flow cytometry
☒	☐	MRI-based neuroimaging

Antibodies

Antibodies used	All suppliers information were described in the main manuscript under 'Materials' section
Validation	Antibodies were validated by manufactures

Eukaryotic cell lines

Policy information about cell lines

Cell line source(s)	Expi293F from ThermoFisher Scientific
Authentication	Cell line was used as per manufacture instruction. No authentication was done prior to usage
Mycoplasma contamination	Cell line were not tested for microplasma contamination
Commonly misidentified lines (See ICLAC register)	NA

March 2021

Human research participants

Policy information about studies involving human research participants

Population characteristics	All ages and genders with positive PCR for COVID-19
Recruitment	Positive COVID-19 results from PCR
Ethics oversight	National University of Singapore

Note that full information on the approval of the study protocol must also be provided in the manuscript.
